# Secretome of the Olfactory Ensheathing Cells Influences the Behavior of Neural Stem Cells

**DOI:** 10.3390/ijms26010281

**Published:** 2024-12-31

**Authors:** Yu-Huan Hsueh, Kuan-Po Chen, Waradee Buddhakosai, Phung-Ngan Le, Ying-Wu Hsiung, Yung-Yi Tu, Wen-Liang Chen, Huai-En Lu, Yuan-Kun Tu

**Affiliations:** 1 Department of Orthopedic Surgery, E-Da Hospital, I-Shou University, Kaohsiung City 824, Taiwan; 2 College of Engineering Bioscience, National Yang Ming Chiao Tung University, Hsinchu City 300, Taiwan; 3 School of Medicine, National Taiwan University, Taipei City 106, Taiwan; 4 Department of Biological Science and Technology, National Yang Ming Chiao Tung University, Hsinchu City 300, Taiwan; 5 Bioresource Collection and Research Center, Food Industry Research and Development Institute, Hsinchu City 300, Taiwan; 6 Institute of Biochemistry and Molecular Biology, National Yang Ming Chiao Tung University, Hsinchu City 300, Taiwan; 7 Center for Regenerative Medicine and Cellular Therapy, National Yang Ming Chiao Tung University, Hsinchu City 300, Taiwan

**Keywords:** olfactory ensheathing cells, secretome, proteome, neuro-regeneration, oxidative stress, reactive oxygen species modulation, neural stem cells, neuronal differentiation, quiescence

## Abstract

Olfactory ensheathing cell (OEC) transplantation demonstrates promising therapeutic results in neurological disorders, such as spinal cord injury. The emerging cell-free secretome therapy compensates for the limitations of cell transplantation, such as low cell survival rates. However, the therapeutic benefits of the human OEC secretome remain unclear. We harvested the secretome from human mucosal OECs and characterized its protein content, identifying 709 proteins in the human OEC secretome from three donors in two passages. Thirty-nine proteins, including neurological-related proteins, such as profilin-1, and antioxidants, such as peroxiredoxin-1 and glutathione S-transferase, were shared between the six samples. The secretome consistently demonstrated potential effects such as antioxidant activity, neuronal differentiation, and quiescence exit of neural stem cells (NSCs). The total secretome produced by OECs protects NSCs from H_2_O_2_-induced reactive oxygen species accumulation. During induction of neuronal differentiation, secretomes promoted neurite outgrowth, axon elongation, and expression of neuronal markers. The secretome ameliorated bone morphogenetic protein 4- and fibroblast growth factor 2-induced quiescence of NSCs. The human OEC secretome triggers NSCs to exit prime quiescence, which is related to increased phosphoribosomal protein S6 expression and RNA synthesis. The human OEC secretome has beneficial effects on NSCs and may be applied in neurological disease studies.

## 1. Introduction

Olfactory ensheathing cells (OECs) are special glial cells that are exclusively present in the olfactory system [1]. During development, OECs differentiate and migrate together with the axons by extending ahead of the axon [2,3,4]. Their primary role is to support olfactory neurons by ensheathing the axon bundles of the olfactory receptor neurons along their path to the glomerular targets of the olfactory bulb in the forebrain. Furthermore, OECs facilitate the continuous renewal of olfactory neurons. OECs are heterogeneous populations [5] that exist in both the peripheral (lamina propria layer of the nasal mucosa) and central (olfactory nerve layer [ONL] of the olfactory bulb) nervous systems with different behaviors [3]. A previous study has suggested that both mucosal OECs and OECs in the ONL of the olfactory bulb can be classified as peripheral glia [6]. Although molecular differences have been identified between OECs in different regions, both mucosal OECs and ONL-OECs possess promising neuro-regenerative effects and promote axon growth, remyelination, and phagocytosis [5,7,8].

OEC transplantation has been demonstrated to be safe in clinical trials; however, the outcomes are variable and affected by several factors, including cell survival rate after transplantation [9]. An animal study showed that the survival rate of transplanted OECs in spinal cord lesions is very low [10]. Recently, cell-free therapies using secretomes or exosomes have been reported to have several advantages over cell therapy, including the ease of manufacturing, storage, and shelf life [11]. The secretome is a collection of biological molecules secreted by cells, and it contributes to cell–cell communication and homeostasis [12]. It comprises soluble proteins such as cytokines, growth factors, and extracellular vesicles, including exosomes [13]. Further, the secretome of adipose-derived mesenchymal stem cells promotes skeletal muscle regeneration through the combined effect of soluble proteins and extracellular vesicles [14]. The secretome of olfactory mucosa mesenchymal stem cells and OECs promoted nervous and muscular regeneration in a rat model of peripheral nerve injury [15]. The secretomes used in these studies were harvested from the conditioned medium. A previous study harvested the total secretome of adipose-derived stem cells by incubating detached cells in phosphate-buffered saline (PBS) at room temperature to eliminate the effect of trace elements in the medium [14]. This method provided the total secretome with the ability to regenerate skeletal muscle. Most studies on the secretome of OECs or olfactory mucosa stem cells have been conducted in animal models. So far, there are no data available regarding how the human OEC secretome impacts NSCs.

Discovering the components of the human OEC secretome is also necessary to better understand the multifaceted role of human OECs and unlock their full potential in repairing neurological injuries. Therefore, this study aimed to (1) characterize the protein content of the total human OEC secretome and (2) observe the effects of the total human OEC secretome on the viability and differentiation of neural stem cells (NSCs).

## 2. Results

### 2.1. Primary OEC Culture and Characterization

**Figure 1:**
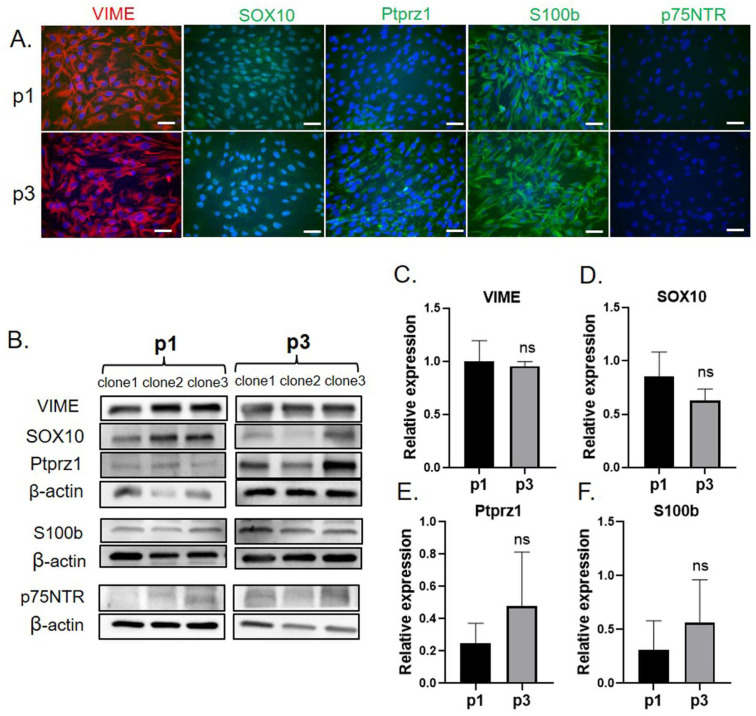
**Characterization of cultured hOECs.** (**A**) Immunocytochemistry staining of OEC markers in cultured cell passage 1 and passage 3. Scale bar = 50 µm. (**B**) Western blot of OEC markers. (**C**–**F**) Densitometry of the Western blot result; vimentin (**C**), SOX10 (**D**), Ptprz1 (**E**), S100b (**F**). ns = not statistically significant.

Primary human OECs were isolated from the lamina propria of the olfactory mucosal tissue and validated using immunocytochemistry and Western blotting. Figure 1A shows representative images of immunocytochemistry targeting OEC-specific markers on the cells from passages 1 and 3, which were the sources of secretomes in this study. Immunocytochemistry results demonstrated that the cultured cells strongly expressed vimentin (VIME) in the cytoplasm of every clone and passage (Figure 1A). SRY-box transcription factor 10 (SOX10) was also expressed in the nuclei of the cells; however, the expression slightly decreased in passage 3 (Figure 1A). The expression of Ptprz1, defined as pan-OECs in a rat model [16], was detected in some areas with very low-intensity signals at passage 1, which increased in passage 3. Western blot analysis also showed the expression of these proteins in all OEC samples (Figure 1B–F). In passage 3, the expression of SOX10 was reduced, whereas that of Ptprz1 and S100b was increased compared with that in passage 1; however, neither of these changes were statistically significant. No significant differences in VIME expression were found between the two passages. Expression of p75NTR was not detected in the cultured cells by either immunocytochemistry or Western blot analysis in passages 1 and 3.

### 2.2. OEC Secretome Proteomic Analysis

**Figure 2:**
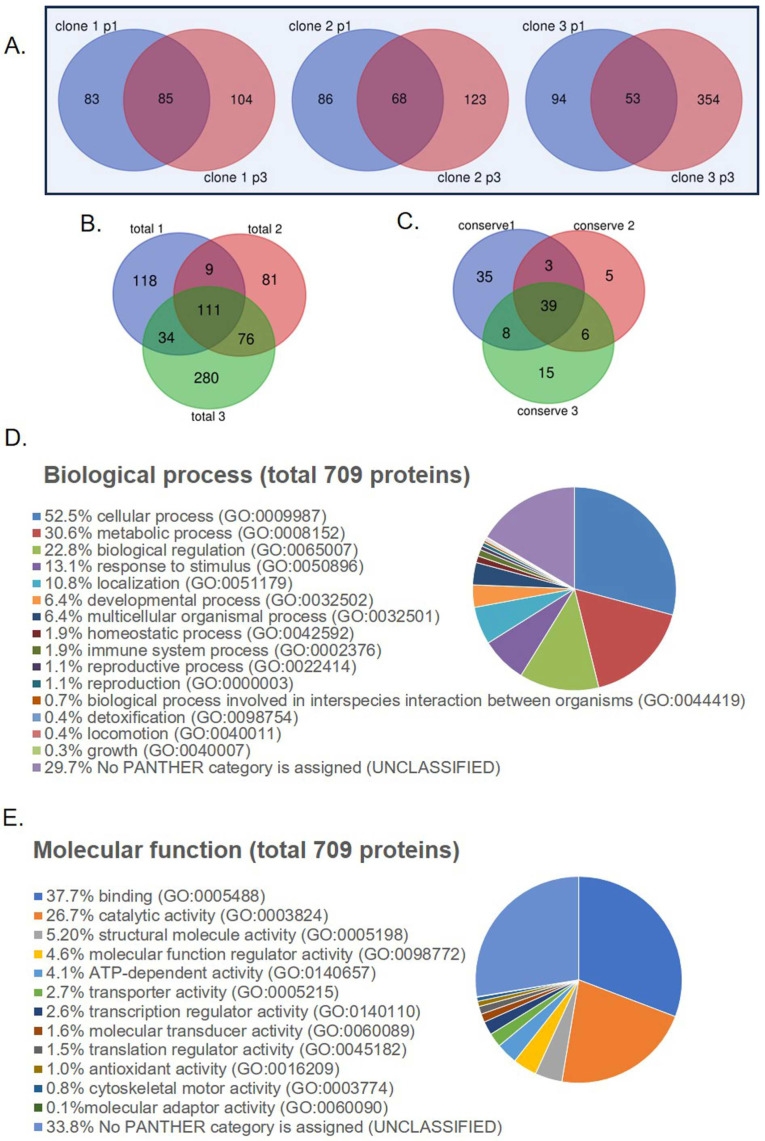
**Protein profile analysis.** (**A**) Venn diagrams showing the mutual hits between two passages of each clone. (**B**) Venn diagram presenting the correlation rate among the total proteins identified in each clone (passage-combination). (**C**) Venn diagram presenting the correlation rate among the conserved proteins found in two passages of each clone. (**D**) GO analysis for biological process and (**E**) molecular function terms of a total of 709 proteins identified in this study.

The total protein concentrations of the secretome from each OEC clone varied from 0.2–0.4 μg/μL. To characterize the protein component of the secretomes from different donors and passages, we analyzed the liquid chromatography with tandem mass spectrometry (LC-MS/MS) proteomic profiles of the secretome from three clones of OECs at passages 1 and 3 using bioinformatics tools. A total of 709 proteins were identified in the six samples. We extracted the conserved proteins found in passages 1 and 3 of each clone (Figure 2A). The numbers of conserved proteins in clones 1, 2, and 3 were 85, 68, and 53, respectively. We found that the number of proteins secreted in passage 3 was higher than that secreted in passage 1 for all clones. This result indicated that the protein content in the secretome of passage 3 was more enriched than that in passage 1. The total protein content of the secretome mixture (combination of passages 1 and 3) was 272, 277, and 501 in clones 1, 2, and 3, respectively. There were 111 mutual hits of the total proteins present in each clone (Figure 2B), indicating that 111 proteins were commonly found in the three secretome mixtures. This suggests that 111 out of the 709 proteins (15.7%) were commonly present among the different clones. Of the 85, 68, and 53 conserved proteins from different passages of clones 1, 2, and 3 (intersected region of each Venn diagram in Figure 2A), only 39 proteins were mutually present in the three different OEC donors, and they were conserved throughout the passages (Figure 2C).

Gene ontology (GO) analysis was performed to categorize the total proteins identified in this study (709 proteins) based on their biological processes or molecular functions. Based on the biological functions, most of the proteins were found to be related to cellular processes (GO:0009987, 52.50%), metabolic processes (GO:0008152, 30.60%), and biological regulation (GO:0065007, 22.80%) (Figure 2D). Based on molecular function, the proteins were mostly classified as binding proteins (GO:0005488; 37.70%) and catalytic activity proteins (GO:0003824; 26.70%) (Figure 2E).

**Figure 3:**
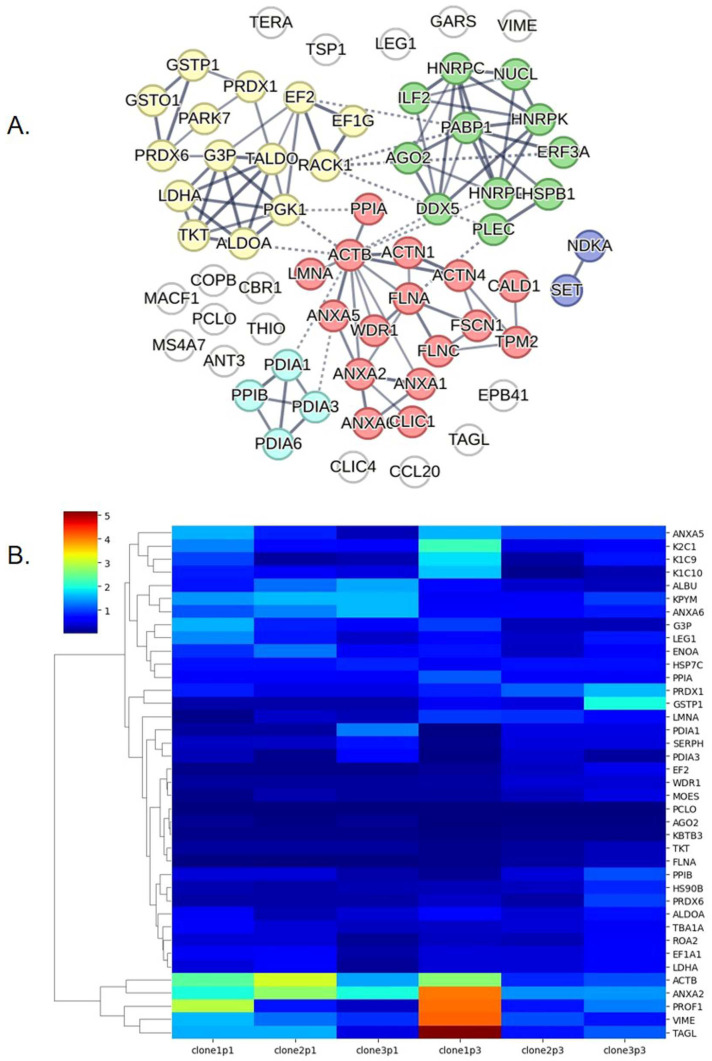
**Protein interaction network and heatmap of protein abundance.** (**A**) Protein–protein interaction network of 111 mutual proteins from passage-combination of each clone calculated using the STRING software 12.0. Proteins were grouped into five major clusters showed in different colors. White nodes are proteins that were not classified into these five clusters. (**B**) Heatmap showing the abundance of 39 conserved proteins found in all six secretome samples.

The protein–protein interaction network of the 111 proteins commonly found in the passage-pooled secretome was analyzed using STRING (Figure 3A, PPI enrichment *p*-value < 10^−16^). The proteins were grouped into interacting clusters using a k-means clustering algorithm. We found that the largest cluster was the actin and actin-binding protein network, and the actin beta chain (ACTB) was the largest hub of this cluster. The second largest network was the catalytic enzyme network, which included peroxiredoxin (PRDX) 1, PRDX6, and other antioxidant proteins such as glutathione S-transferase Pi 1 (GSTP1), glutathione S-transferase omega-1 (GSTO1), and protein/nucleic acid deglycase DJ-1 (PARK7). This finding is consistent with the results of the GO term (molecular function) analysis. Our findings suggest that the passage-pooled secretome mixtures of each donor carried actin-binding and antioxidant-related proteins.

A heatmap representing the abundance of 39 mutual proteins in the six secretome samples was constructed (Figure 3B). Clone 1 secreted proteins at a higher abundance rate than the other two clones. Some proteins, including pyruvate kinase (KPYM), annexin (ANXA) 6, and ACTB, were highly enriched at passage 1 and downregulated at passage 3. Meanwhile, the abundance of some proteins, such as PRDX 1 and GSTP1, increased at passage 3. ACTB and ANXA2 were the most abundant proteins identified in the present study. ACTB, ANXA2, Profilin-1 (PROF1), VIME, and transgelin (TAGL) abundance were particularly high in clone 1. The list of total proteins identified in this study is provided in Supplementary Table S1.

### 2.3. OEC Secretome Ameliorates Reactive Oxygen Species (ROS) Accumulation in NSCs

**Figure 4:**
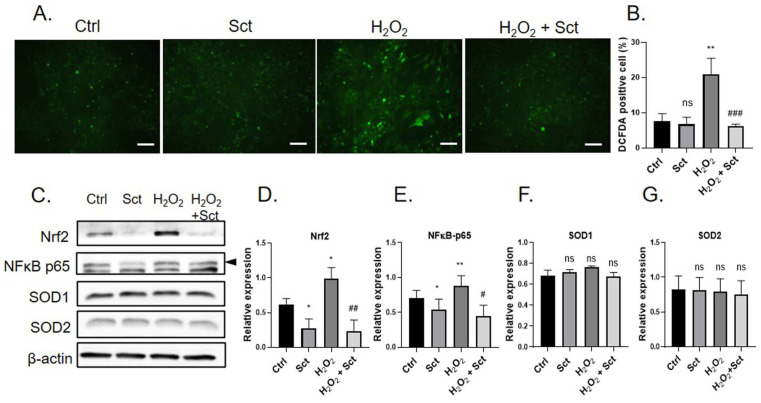
**hOEC secretome exhibited antioxidation ability.** (**A**) DCFCA staining in NSCs. Scale bar = 100 µm. (**B**) Quantification of DCFDA staining result showing the percentage of DCFDA-positive cells. (**C**) Western blot analysis image. (**D**–**G**) Densitometry analyses results. * significant difference compared to the control group (* *p* < 0.05, ** *p* < 0.01), # significant difference compared to the H_2_O_2_ group (# *p* < 0.05, ## *p* < 0.01, ### *p* < 0.005), ns = not statistically significant.

Neuronal cells are sensitive to oxidative stress, which has been implicated in several neurological diseases [17]. In this study, the mutual hits identified in the proteomic analysis included antioxidant proteins. Therefore, we investigated the antioxidative effects of the OEC secretome on NSCs. NSCs were pre-treated with H_2_O_2_ to induce ROS accumulation before secretome treatment. Secretomes from different passages were pooled into a secretome mixture for each donor and used for each treatment. A significant increase in the number of ROS-positive cells in the H_2_O_2_-treated group was revealed by 2′,7′-dichlorofluorescin diacetate (DCFDA) (Figure 4A,B). Secretome-treated cells showed significantly fewer positive cells than the H_2_O_2_ group. Expression levels of oxidative stress-related proteins, including superoxide dismutase (SOD) 1, SOD2, nuclear factor erythroid 2-related factor 2 (Nrf2), and nuclear factor–kappa B (NFκB), were determined using Western blotting. We observed that the expression of Nrf2 and NFκB was significantly increased upon H_2_O_2_ treatment. This was significantly ameliorated by secretome treatment (Figure 4C–E). The expression of the antioxidant proteins SOD1 and SOD2 were not affected (Figure 4C,F,G). This result indicates that the OEC secretome has an antioxidant effect on H_2_O_2_-induced ROS accumulation and regulates Nrf2 and NFκB expression in NSCs.

### 2.4. OEC Secretome Stimulated Exit of Quiescence in NSCs at the Molecular Level

**Figure 5:**
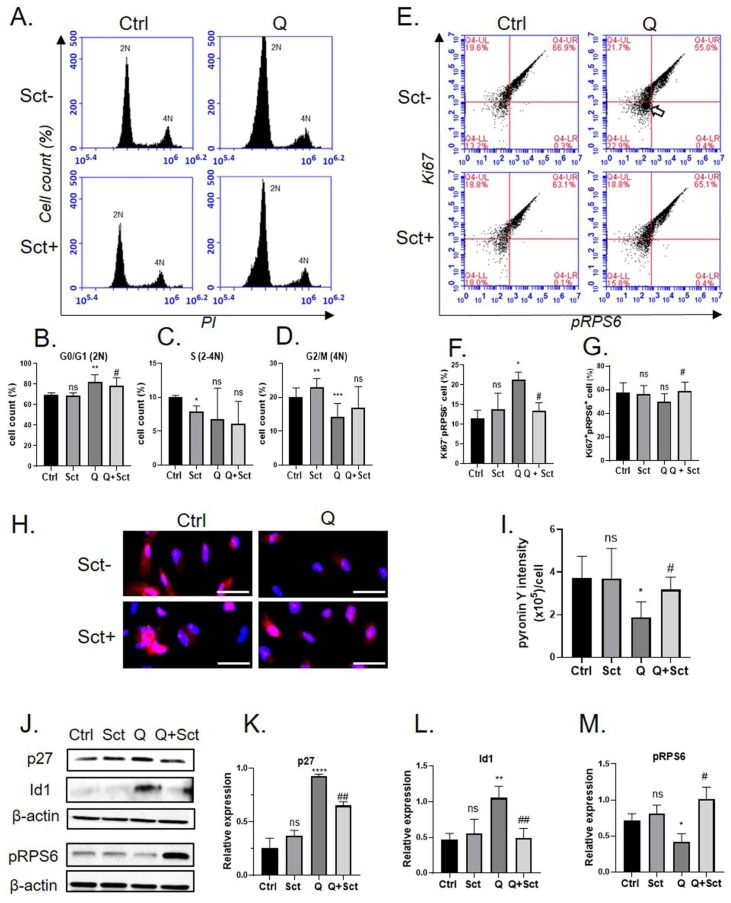
**hOEC secretome affected the quiescence state of NSCs.** (**A**) Propidium iodide staining of NSCs in each experimental group. (**B**–**D**) Bar graph showing cell population in each cell cycle phase compared between the experimental groups. (**E**) Flow cytometry result of Ki67/pRPS6-labeled NSCs in different groups. (**F**,**G**) Quantification of Ki67/pRPS6-labeled NSCs. (**H**) Pyronin Y staining result of NSCs. Scale bar = 100 µm. (**I**) quantification of pyronin Y staining. (**J**) Western blot analysis images representing quiescence markers. (**K**–**M**) Densitometry of Western blot analysis. * significant difference compared to the control group (* *p* < 0.05, ** *p* < 0.01, *** *p* < 0.005, **** *p* < 0.001), # significance difference compared to the Q group (# *p* < 0.05, ## *p* < 0.01), ns = not statistically significant.

Although NSCs reside in the adult brain, most of them are inactive and exist in reversible cell cycle exits or quiescent states [18,19]. We sought to determine whether the OEC secretome affects the quiescent NSC. We induced prime quiescence in NSCs by incubating the cells with bone morphogenetic protein 4 (BMP4) and fibroblast growth factor 2 (FGF2), as described previously [20]. For cell cycle analysis, the cells were stained with propidium iodide and analyzed using flow cytometry (Figure 5A). The results showed no significant difference in the 2N population, which represents cells in the G1 or G0 phase, between the untreated control and secretome-only treated groups (Figure 5B). The secretome-only treatment increased the 4N (G2/M phase) population while decreasing the number of 2-4N (S phase) cells (Figure 5C,D). We found that the 2N population was significantly increased after BMP4/FGF2 treatment (Q; quiescent cells) and was reversed upon secretome treatment (Figure 5B). Meanwhile, the number of cells in the 4N (G2/M) phases in the Q group was significantly reduced. Secretome treatment reversed this effect by insignificantly increasing G2/M cell populations (Figure 5D). This result indicates that BMP4/FGF2 induced G1/G0 cells in human NSCs and that the OEC secretome regulated the cell cycle of NSCs by inducing G1/G0 phase exit and stimulating cells to reenter the cell cycle.

To distinguish quiescent cells (G0) from active-cycling cells (G1), a previous study used Ki67 and ribosomal protein subunit 6 (RPS6) expression and beta-galactosidase staining [21]. To rule out senescence, we first performed senescence-associated beta-galactosidase staining in each experimental group. No increase in the staining signal was observed in the BMP4/FGF2 and/or secretome treatment groups. The consistency and expression of p21, a senescence marker, were not detected via Western blotting (Supplementary Figure S1). The cells were then sorted using flow cytometry for Ki67/pRPS6 protein expression, and the populations of actively proliferating (Ki67+/pRPS6+) and quiescent cells (Ki67-/pRPS6-) in each group were compared (Figure 5E). The result showed that the number of quiescent cells or Ki67-/pRPS6- cells significantly increased in the BMP4/FGF2 treated groups (thick white arrow in the figure). However, this was significantly reversed in the secretome-treated group (Figure 5F). Ki67+pRPS6+ cells, representing active-cycling cells, decreased in quiescent cells and significantly increased upon secretome treatment (Figure 5G). This result indicates that the OEC secretome promotes NSC exit from the quiescence stage induced by BMP4/FGF2 by stimulating Ki67 and pRPS6 expression.

The cells in the G1 and G0 phases contain equal DNA content; however, G0 cells contain low RNA content owing to reduced transcriptional activity [22]. Thus, to confirm the quiescence of G0 cells, we further observed the RNA content in the cells using pyronin Y staining. As depicted in Figure 5H,I, the RNA content of normal NSCs was not affected by secretome treatment. BMP4/FGF2-induced quiescent cells showed a significant reduction in pyronin Y levels. However, the pyronin Y signal in Q cells increased in the secretome-treated groups. This result indicates that the OEC secretome promoted RNA synthesis in protein-induced quiescent cells.

We analyzed the expression of quiescent marker proteins p27 and Id1 under different conditions (Figure 5J). The protein expression of p27 and Id1 was significantly induced by BMP4/FGF2 treatment (Figure 5K,L). Treatment with OEC secretome ameliorated this aberrant expression. The pRPS6 protein expression was validated using Western blotting. The results demonstrated that NSCs under BMP4/FGF2-induced quiescence condition significantly increased the expression of pRPS6 (Figure 5M). Overall, these results indicate that BMP4/FGF2 protein treatment can induce the prime quiescence phase of NSCs and that the OEC secretome supports quiescence exit at the molecular level.

### 2.5. OEC Secretome Enhanced Neuronal Differentiation from iPSC-Derived NSCs

**Figure 6:**
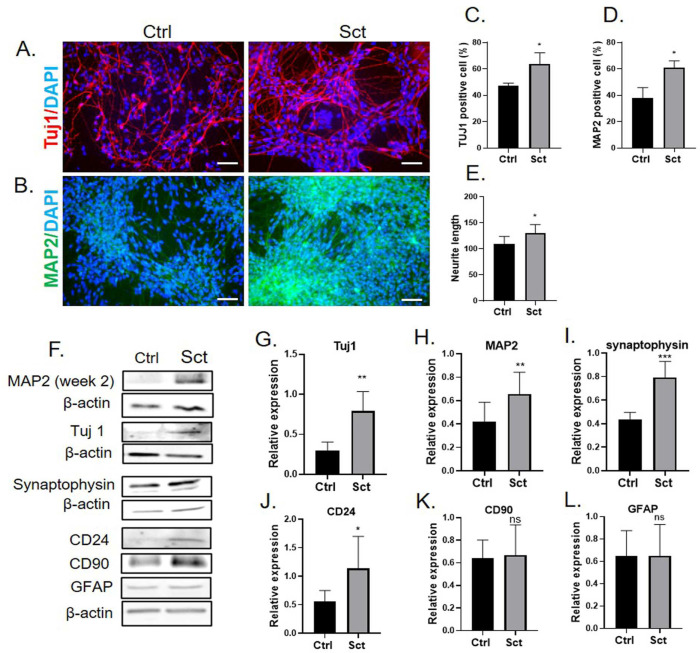
**hOEC secretome enhances neuronal differentiation of NSCs.** (**A**,**B**) Immunocytochemistry targeting neuron markers TUJ1 (**A**) and MAP2 (**B**) in control and secretome-treated NSCs. Scale bar = 50 µm. (**C**,**D**) Bar graphs showing the percentage of TUJ1- (**C**) and MAP2-positive (**D**) cells quantified using ImageJ. (**E**) Neurite length quantified using the NeuriteQuant software. (**F**) Western blot analysis of neuron protein expression. (**G**–**L**) Densitometry of Western blot analysis results. * significant difference compared to the control group (* *p* < 0.05, ** *p* < 0.01, *** *p* < 0.005), ns = not statistically significant.

To examine the effects of the OEC total secretome on the neuronal differentiation of NSCs, we performed an in vitro neuronal differentiation assay with and without the secretome. Total secretomes from different donor OECs were added to the cells on day 1 after seeding. Morphological changes and neurite growth were observed on day 7. Immunocytochemistry for detecting neuron-specific class III β-tubulin (TUJ1) and microtubule-associated protein 2 (MAP2) was performed to identify differentiated cells (Figure 6A,B). We found that MAP2 expression in the neurites was very low in the first week and was detectable under a fluorescence microscope in the second week of differentiation (Figure 6B). The percentage of TUJ1- and MAP2-positive cells was counted (Figure 6C,D), and the neurite length was calculated from TUJ1 staining using NeuriteQuant software 1.23 and compared between the no-treatment control and secretome treatment groups (Figure 6E). The result showed that the numbers of TUJ1- and MAP2-positive cells were significantly higher in the secretome than in the control group. The neurite length in the secretome-treated cells was also significantly longer than that in the control cells. The expression levels of neuronal markers were analyzed using Western blotting, and the results are shown in Figure 6F. The expression levels of neuronal cell markers such as TUJ1, MAP2, and synaptophysin were significantly increased in secretome-exposed cells (Figure 6G–I). The protein level expressions of a neuronal marker (CD24), an oligodendrocyte marker (CD90), and astrocyte marker glial fibrillary acidic protein (GFAP) were also observed based on a previous report [23]. The expression level of CD24 was higher in the secretome group than in the control group (Figure 6J). We found no change in GFAP expression. Meanwhile, the expression of CD90 changed but this did not reach the level of significance and was inconsistent among NSC clones (Figure 6K,L). These results indicate that the total secretome of OECs stimulates neural differentiation of NSCs by enhancing the expression of neuron-enriched proteins and promoting neurite elongation.

## 3. Discussion

Previous studies have used different strategies for isolating human OECs [24,25,26]. Although these methods have a high success rate, there are controversial issues related to specific markers for OEC. Voronova et al. [24] and Li et al. [25] showed p75NTR expression in cultured human OECs. A study on the olfactory tissue from human fetuses showed that human OECs do not express p75NTR, but Schwann cells and perineurial olfactory nerve fibroblasts do [27]. The study suggested S100/VIME/SOX10 as markers of human fetal OECs, which is consistent with the findings of the present study. Furthermore, in a study involving mice [16], a novel OEC marker, Ptprz1, encoding a receptor-like tyrosine phosphate was identified. In the present study, we found that cultured OECs expressed Ptprz1 using Western blotting and immunocytochemistry. Although its expression in passage 1 was low, it increased in passage 3. To the best of our knowledge, no previous reports have suggested Ptprz1 expression in human OECs and this is the first study to reveal the expression of VIME/S100/SOX10/Ptprz1 in primary cultured human OECs.

Moreover, this is likely the first study to report the proteomic profile of the human OEC secretome harvested in PBS. We identified novel proteins in the total secretome, some of which have been previously reported for their neurogenic and neuroprotective functions. A previous study reported the expression of neural regeneration-related proteins, including hevin, secreted protein acidic and rich in cysteine (SPARC), and PFN1 (or PROF1), in the secretome of OECs isolated from newborn rat olfactory bulbs [28]. In line with this, we also detected PROF1 in all human OEC secretome samples in this study, even though the protein abundance levels varied among samples. Hevin and SPARC, which are enriched in the rat OEC secretome, were not detected in the present study. However, we identified ANXA2, ANXA6, galectin-1 (LEG1), tubulin alpha chains (TBA), and filamin A (FLNA). TBA1A, LEG1, and FLNA, which are found in low abundance in all samples, were reported for their beneficial effects on cellular processes and neurogenic or neuroprotective effects. Tubulin (both TBA and TBB) is a major component of microtubules, which are important for migration and differentiation of neurons [29]. It also plays a pivotal role in cell viability, neurite outgrowth, and protection of neurons from ROS [30]. It is highly expressed during neuronal process extension and may regulate cytoskeletal remodeling during nerve regeneration [31]. LEG1 is a microglial regulator that targets p38MAPK, CREB, and the NF-κB pathway, attenuates pro-inflammatory cytokines [32,33], and reduces oxidative stress [34]. By attenuating microglial activity, LEG1 ameliorates neurodegeneration in in vitro and in vivo Parkinson’s disease models [35]. Transplantation of LEG1-overexpressed NSCs into the ischemic brain of mice reduced infarct volume [33]. Furthermore, LEG1 has been identified as an important factor that promotes neural crest cell generation [36] and the proliferation of adult NSCs [37]. FLNA, an actin-binding protein, regulates neuronal migration, and interacts with partner proteins to orchestrate ion channels of neurons [38]. It also regulates neural progenitor proliferation, and the loss of FLNA interrupts the cell cycle by impairing the G2 to M phase transition, which leads to cell cycle prolongation of neural progenitors [39].

Based on the protein–protein interaction network analysis, many of the highly abundant proteins identified in this study (actin, annexin A2 [ANXA2], PROF1, VIME, and TAGL) were related to the actin-binding network. Actin and actin-binding proteins (ABPs) are abundant in the neuronal cytoskeleton. They play important roles in axonal elongation, branching, guidance, and synapse formation. These proteins are also related to filopodia attenuation and the formation of dendritic spines and synapses [40]. A report revealed that PFN1 and PFN2 (PROF2) contribute to brain development [41]. PFN1 was demonstrated to be an axonal growth promoter through actin-microtubule coordination. It promoted actin retrograde flow and microtubule growth in the growth cone of the axon in a formin-dependent manner. PFN1 ablation limited axon regeneration after spinal cord and sciatic nerve injury [42]. TAGL was one of the most abundant proteins found in a single donor. It is a member of the calponin family of ABPs and is expressed in OECs but not Schwann cells [43,44]. TAGL overexpression reduces the proliferation of human bone marrow-derived stromal stem cells while enhancing cell migration and differentiation toward adipocytic and osteoblastic lineages [45]. To date, there is no evidence of the neurological function of TAGL. ANXA2 plays a major role in multiple cellular processes, including exocytosis and endocytosis [46]. Downregulation of ANXA2 interferes with cell division and proliferation [47]. ANXA2 is also expressed in neurons, distributed in axonal branches during the growth and extension of synaptic networks, and is increased by the depolarization of neurons [48]. It is enriched in sensory neurons and regulates nociceptive signaling in vertebrates [49]. ANXA2 and ANXA6 interact with tau protein, a member of the tau/MAP2/MAP4 family. This binding contributes to axon localization of tau, which is important for neuronal development [50].

Overall, the findings of these previous studies and the results of the present study regarding tubulin protein (TBA), actin, PROF1, ANXA2, and ANXA6 in the secretome lead us to hypothesize that the OEC secretome likely enhances axon elongation during neuron differentiation through the interaction of these secretome protein contents, such as profilin–actin binding or the ANXA2-tau/MAPs pathway. Notably, this study only presents the detectable protein profile of the human OEC secretome, and shows that the neuron differentiation-promoting effect is influenced by the secretome. Further studies focusing on the neurogenic and neuro-regenerative effects, as well as the clinical applications of these target proteins, are warranted.

We also identified three important antioxidant enzymes: PRDX1, PRDX6, and GSTP1. A previous study has revealed that although high intracellular ROS levels under oxidative stress can potentially damage cells, low levels of ROS are required for cellular processes, including stem cell proliferation and differentiation [51]. To reduce and maintain low levels of endogenous peroxides, PRDX reacts with H_2_O_2_ by oxidizing cysteine residues [52]. PRDX1 scavenges ROS in cells under oxidative stress [53] and is required for the antioxidant function of stroke-associated microglia, which are specifically activated during stroke ischemia/reperfusion injury. The deficiency of PRDX1 exacerbated microglial cell death and brain damage in ischemic stroke [54]. PRDX6 has also been implicated in ROS regulation [55,56]. Similarly, several studies have revealed that GSTP1 protects cells from oxidative stress by reducing ROS production, and loss of GSTP1 increases intracellular ROS production [57,58]. Even though SOD1 and SOD2, important enzymes that control ROS levels, were not significantly changed by the OEC secretome, the secretome treatment reduced the expression of Nrf2 and NFκB, two key regulators of oxidative stress response and inflammation in the cells. A previous study showed that GSTP interacts with Nrf2 and controls both Nrf2-dependent and independent cell responses to alkylating agents [59]. In the present study, we only demonstrated the antioxidant effects of OEC secretomes through the H_2_O_2_-induced ROS model. Based on previous reports and our result, we hypothesized that GSTP and/or PRDX found in OEC secretomes may play a role in controlling Nrf2 and NFκB expression levels and function. However, a comprehensive study is required to clarify our hypothesis. Given that the metabolic imbalance and aberrant ROS accumulation are related to several neurological diseases and neurodegeneration [60], our study showed that the OEC secretome, by its antioxidant properties, may be therapeutically exploited for the treatment of such diseases. However, further validation is required in this regard.

The total number of identified proteins varied in the present study; for instance, clone 3 had a predominantly higher number than that of the other donors. Meanwhile, clone 1 contained the least variety of proteins but produced the highest concentration of abundant proteins. The donor-specific factors such as sex and age may contribute to various cellular functions and secretory properties [61]. To verify the effect of these factors, more OEC samples from donors of different ages and both sexes should be harvested for proteomic study. Although we characterized the secretomes from three different donors and two passages, only 39 proteins were commonly present in these secretomes. However, the experimental results were similar among the three secretome mixtures (low standard deviation). Therefore, we anticipate that the high abundance of common proteins among the 39 proteins may play a pivotal role in these therapeutic outcomes and will be valuable for further studies.

Although NSCs are a source of neurons and reside in the brains of adult mammals, the majority of these adult NSCs undergo reversible cycle exits or quiescence [18]. The quiescent state of adult stem cells involves metabolic changes that maintain low levels of transcription and translation [62]. One of the widely used criteria to discriminate quiescent from senescent and stressed cells is to evaluate the expression of Ki67, ribosomal protein (RPS6), and beta-galactosidase [21,63]. This is the first study to observe the role of the human OEC secretome in the cell cycle and quiescence of NSCs using the aforementioned criteria. A combination of BMP4 and FGF2 was used to prime the quiescent state induction in mouse brain cells in a previous study [20]. However, this dose produced unsatisfactory results in our study. Therefore, we applied this method with minor modifications (reduced dose of FGF2) to induce quiescence in human-induced NSCs. The quiescence markers, Id1 and p27, showed significant changes. BMP4/FGF protein combination also increased the G1/G0 phase of the cell cycle. Together with the Ki67-phospho (p) RPS6- cell count, we speculated that this protein combination treatment also induced prime quiescence in human NSCs, probably through a different mechanism from that in mouse cells. While we found that the OEC secretome regulates protein-induced prime quiescence in NSCs, as demonstrated by Ki67/pRPS6, pyronin Y, and protein expression analyses, the response to the OEC secretome of normal proliferating cells seemed to vary from no effect to slightly increased quiescent phenotypes of NSCs. Although the OEC secretome alleviated the expression of quiescent proteins in the induced prime quiescent state, we observed only a subtle increase in the G2/M phases after treating the induced quiescent NSCs with the OEC secretome. Therefore, we hypothesized that the human OEC secretome in this study has a molecular effect on quiescence exit but does not predominantly induce cell cycling and proliferation. The regulatory mechanisms behind this quiescent exit and the effect of the OEC secretome on NSC proliferation are presently unknown and remain to be clarified.

A previous report in rats revealed that soluble factors from OECs promote proliferation and inhibit the neuronal differentiation of neural progenitor cells [64]. This finding is inconsistent with those of the present study. We found that the OEC secretome enhanced neuronal differentiation by stimulating neuronal proteins and promoting neurite elongation. Although we did not observe a change in CD90 and GFAP in this study, we still lack evidence to clearly classify the differentiation lineage of NSCs. Further studies investigating the expression of other glial lineage, including oligodendrocyte and astrocyte, markers during differentiation will be required to provide more comprehensive information. We also observed that the neurites of neuronal cells differentiated in the medium supplemented with the OEC secretome were better arranged than those in the control group. A similar issue has been reported in a previous study [4], which showed that olfactory axons co-cultured with mucosal OECs were less dispersed than those co-cultured with ONL OECs. This phenomenon may be related to the characteristics of mucosal OECs, which likely mediate the sorting and guidance of olfactory nerve axons. They adhere to each other and promote cell-cell adhesion and the extension of axons [65]. Cell-cell adhesion between OECs is related to axon fascicle formation [5].

To summarize, proteomic profiles of human mucosal OECs harvested in PBS revealed interesting elements related to neurogenesis and neuroprotective function, such as PROF1, ANXA, LEG1, TBA, TBB, and some antioxidant enzymes, including PRDX and GSTP1. We demonstrated that the secretome of human OECs has an antioxidant effect and reduces intracellular ROS overproduction in NSCs. In normally proliferating NSCs, the OEC secretome appears to maintain stemness by regulating cell quiescence and differentiation. In the induced quiescent state, the secretome induces quiescent NSCs to reenter the cell cycle or active state. During differentiation, the secretome enhances neuronal differentiation of NSCs by enhancing the expression of neuronal markers and facilitating neurite outgrowth and elongation. Therefore, human mucosal OEC secretome harvesting in this scenario may be a potential source for neurological therapy in the future. However, further in vivo validation is required to confirm these results, and the mechanisms by which the secretome regulates these biological processes are yet to be clarified.

## 4. Materials and Methods

The specific data for all materials used in this study are included in Supplementary Table S2.

### 4.1. Cell Culture

We isolated OECs from human olfactory mucosa (OM) tissues, which were donated by three unrelated donors: one female donor (24 years old) and two male donors (28 and 58 years old). The sample collection protocol and conceptual research framework were approved by our Institutional Ethical Committee (Project Identification Code: EMRP44109N; Date of Approval: 15 December 2020; Ethics Committee: E-Da Hospital Institutional Review Board). Informed consent was documented and signed by the donors prior to the surgery. Under anesthesia, an endoscope was used to facilitate sample collection. OECs were isolated as previously described [24]. Briefly, the OM was washed three times in PBS. The olfactory epithelium (thin, translucent layer) was dissected and cleared under a microscope. The lamina propria (thick layer) was placed in a culture dish containing 1.89 U/mL dispase II neutral protease (Gibco^TM^, Life Technologies, Grand Island, NY, USA) and cut into small pieces using sterile scissors. The sliced tissue was then incubated for 20 min in a 37 °C incubator. Dissociated cells were pelleted by centrifuging the sample suspension at 200× *g* for 3 min. The supernatant was decanted. The pellet was then resuspended in the culture medium containing Dulbecco’s modified Eagle’s medium: Nutrient Mixture F12 (DMEM/F12) (Gibco^TM^, Life Technologies, Grand Island, NY, USA), supplements, penicillin-streptomycin (Gibco^TM^, Life Technologies, Grand Island, NY, USA), and cultured at 37 °C with 5% CO_2_. The culture medium was replaced with fresh media every 2–3 days.

In this study, two clones of induced pluripotent stem cells (iPSCs) reprogrammed from peripheral blood mononuclear cells using the Sendai Reprogramming Kit were used to generate NSCs. iPSCs were plated on a culture dish pre-coated with Matrigel^®^ (Corning^®^, Bedford, MA, USA) and maintained in StemFlex^TM^ medium (Gibco^TM^, Life Technologies, Grand Island, NY, USA). To induce NSC differentiation, the PSC Neural induction medium (Gibco^TM^, Life Technologies, Grand Island, NY, USA) was used following the manufacturer’s instructions. Briefly, the iPSC colonies were detached, replated onto new Matrigel-coated dishes, and incubated in the iPSC culture medium for 24 h. The medium was then replaced with PSC Neural induction medium, and the cells were cultured for 7 days with refreshing of the medium every 2 days. Subsequently, passage 0 NSCs were harvested and subcultured to passage 1, which was maintained in the neural expansion medium (neural induction medium plus DMEM/F12).

### 4.2. OEC Secretome Generation

hOECs (passages 1 and 3) were detached with trypsin and centrifuged at 300× *g* for 3 min, followed by two washes with PBS. OECs were aliquoted with 1 × 10^6^ cells per 400 μL PBS and maintained overnight at room temperature. On the second day, the supernatant was collected and filtered through a 0.22 μm membrane. After that, it was centrifuged at 2000× *g* for 20 min at 4 °C. The solution was collected as the total secretome and stored at −80 °C for further use. For the proteomic analysis, each sample was characterized separately. For the in vitro experiments, secretomes from different passages were pooled (1:1 ratio) as the secretome mixture of each donor.

### 4.3. Western Blotting

Total protein was isolated for Western blot analysis by incubating the cells with lysis buffer (Pierce^®^ RIPA buffer, Thermo Scientific, Rockford, IL, USA) for 20 min at 4 °C with intermittent vortexing followed by centrifugation at 13,000× *g* for 10 min. The protein quantification was performed using bicinchoninic acid assay (BCA). The total protein (20 or 30 µg for low-expressed protein detection) was resolved using sodium dodecyl-sulfate polyacrylamide gel electrophoresis (SDS-PAGE) and transferred onto a polyvinylidene fluoride membrane. The membranes were then incubated in a blocking solution containing 5% skim milk in Tris-buffered saline + 0.1% Tween 20 (TBST) at room temperature for 1 h. The membrane was then incubated overnight with primary antibodies at 4 °C. Primary antibodies used in this study were anti-VIME (1:1000), anti-SOX10 (1:1000), anti-Ptprz1 (1:2000), anti-S100 (1:500), anti-p75 NGF (1:1000), anti-Nrf2 (1:1000), anti-NFκBp65 (1:1000), anti-SOD1 (1:1000), anti-SOD2 (1:1000), anti-CD9 (1:1000), anti-p27 Kip1 (1:1000), anti-ID1 (1:1000), anti-MAP2 (1:1000), anti-beta III tubulin (1:1000), anti-Synaptophysin (1:1000), anti-CD90 (1:1000), and anti-cluster of differentiation 24 (CD24; 1:1000). After incubation with the primary antibody, the membranes were washed thrice in TBST for 5 min each and incubated with HRP peroxidase-conjugated anti-rabbit or anti-mouse IgG at room temperature for 1 h on a rocker. The signal was detected using the enhanced chemiluminescence reagent and observed in a chemiluminescence imaging system (GeneGnome XRQ; Syngene, Cambridge, UK).

### 4.4. Sample Preparation and Proteomic Analysis

The total protein concentration of the hOEC secretome was evaluated using BCA assay (Pierce^TM^ BCA Protein Assay Kits, Thermo Scientific, Rockford, IL, USA) before subjecting to SDS-PAGE electrophoresis. The gel was stained with Coomassie blue with gentle agitation and a brief rinse. Individual gel bands were carefully excised and transferred to microcentrifuge tubes containing sterile water for mass spectrometry. Protein was in-gel digested with trypsin, purified, and desalted. LC/MS/MS was performed by Mission Biotech Inc. Raw data were analyzed using the MASCOT software 2.8 to identify proteins based on the peptide profile. GO term analysis of the identified proteins was conducted using PANTHER 18.0 software. Protein–protein interactions were analyzed using the STRING 12.0 software. A heat map showing the protein abundance in each secretome sample was generated at https://www.kaggle.com/ (accessed on 27 October 2023).

### 4.5. Immunocytochemistry

For hOEC characterization, hOECs at a density of 1 × 10^5^ cells/mL were plated in 48- or 96-well plates. Cells were cultured in the complete medium to reach 80% confluency. The cells were washed with PBS and fixed in 4% paraformaldehyde for 10–15 min. After three washes with PBS, the cells were incubated in a blocking solution containing 3% bovine serum albumin (BSA) and 0.1% triton X-100 in PBS for 30 min at room temperature. Then, the cells were incubated overnight with primary antibodies in 1% BSA at 4 °C. The cells were washed three times with PBS and incubated for 1 h with secondary antibodies (goat anti-rabbit Alexa Fluor 488 or goat anti-mouse Alexa Fluor 594). Cell nuclei were counterstained with a DAPI mounting medium. The images were acquired using an inverted fluorescence microscope (Olympus CKX53, Olympus, Hachioji, Tokyo, Japan). The antibodies used for immunocytochemistry in this study were OEC markers such as S100β (1:200), VIME (1:300), p75NTR (1:200), and SOX10 (1:200). Additionally, antibodies against Tuj1 (1:300) and MAP2 (1:300) were used for neuronal labeling after neuronal differentiation.

### 4.6. Induction of Oxidative Stress and DCFDA

NSCs at a density of 1 × 10^4^ cells/100 µL medium were seeded into each well of a 96-well plate pre-coated with Geltrex^TM^ (Gibco^TM^, Life Technologies, Grand Island, NY, USA). To induce oxidative stress, cells were incubated in 100 µM of H_2_O_2_ for 1.5 h at 37 °C. The cells were then treated with 10 ng/mL hOEC-sct or an equal volume of PBS (control) and incubated for 24 h. The reactive oxygen species (ROS) activity of NSCs was evaluated using a DCFDA—Cellular ROS assay kit (Abcam, Cambridge, UK) following the manufacturer’s instructions. The green fluorescent signal was observed and captured using a fluorescence microscope. The percentage of positive ROS signals was calculated from the images using the ImageJ 1.53e software (National Institutes of Health, Bethesda, MD, USA).

### 4.7. Quiescence Induction

To induce the prime quiescent state in NSCs, we followed a method published earlier [20] with minimal modifications. Briefly, 1 × 10^5^ cells/mL NSCs were cultured to reach 70–80% confluency. Subsequently, the supplement was withdrawn to induce quiescence. NSCs were exposed to 10 ng/mL rhBMP-4 and 5 ng rhFGF for 48 h. Afterward, the cells were incubated with 10 mg/mL of OEC secretome for 24 h. An equal volume of PBS was used as the control. The cells were harvested for protein analysis using Western blotting, flow cytometry for Ki67/pRPS6, and cell cycle analysis.

### 4.8. Neuronal Differentiation

The cell culture dish was pre-coated with poly-D-lysine (Gibco^TM^, Life Technologies, Grand Island, NY, USA) for 1 h at room temperature, followed by laminin (Gibco^TM^, Life Technologies, Grand Island, USA) for 1 h in a 37 °C incubator. NSCs in passages 1–4 were subcultured at a cell density of 250,000 cells/mL in the differentiation medium containing CultureOne^TM^ supplement (Gibco^TM^, Life Technologies, Grand Island, NY, USA), following the manufacturer’s instructions. Half of the cell culture medium was replaced with fresh medium every 2 days. Differentiated cells were processed for Western blot analysis or immunocytochemistry for detecting neuronal markers such as TUJ1 and MAP2. TUJ1- and MAP2-positive cells were counted from the fluorescence images using the ImageJ software. The neurite length was quantified using NeuriteQuant 1.23 [66].

### 4.9. Statistical Analysis

Statistical analyses were performed using GraphPad Prism v.8.0.1 (GraphPad Software, Boston, MA, USA). A two-tailed Student’s *t*-test was used to compare two groups, and a one-way analysis of variance was used to compare multiple experimental groups. Tukey’s post hoc analysis was used for multiple comparisons. A 95% confidence interval was used to determine significance. Differences were considered significant if *p* < 0.05 (* or #), *p* < 0.01 (** or ##), *p* < 0.005 (*** or ###), or *p* < 0.001 (**** or ####).

## Data Availability

The original contributions presented in this study are included in the article/Supplementary Materials. Further inquiries can be directed to the corresponding authors.

## Supplementary Materials

The supporting information can be downloaded at: https://www.mdpi.com/article/10.3390/ijms26010281/s1.
