# Supplementary figures and images for: Secretome of the Olfactory Ensheathing Cells Influences the Behavior of Neural Stem Cells
Source: Int J Mol Sci. 2024 Dec 31;26(1):281. doi: 10.3390/ijms26010281 (PMC11720278; doi:10.3390/ijms26010281)

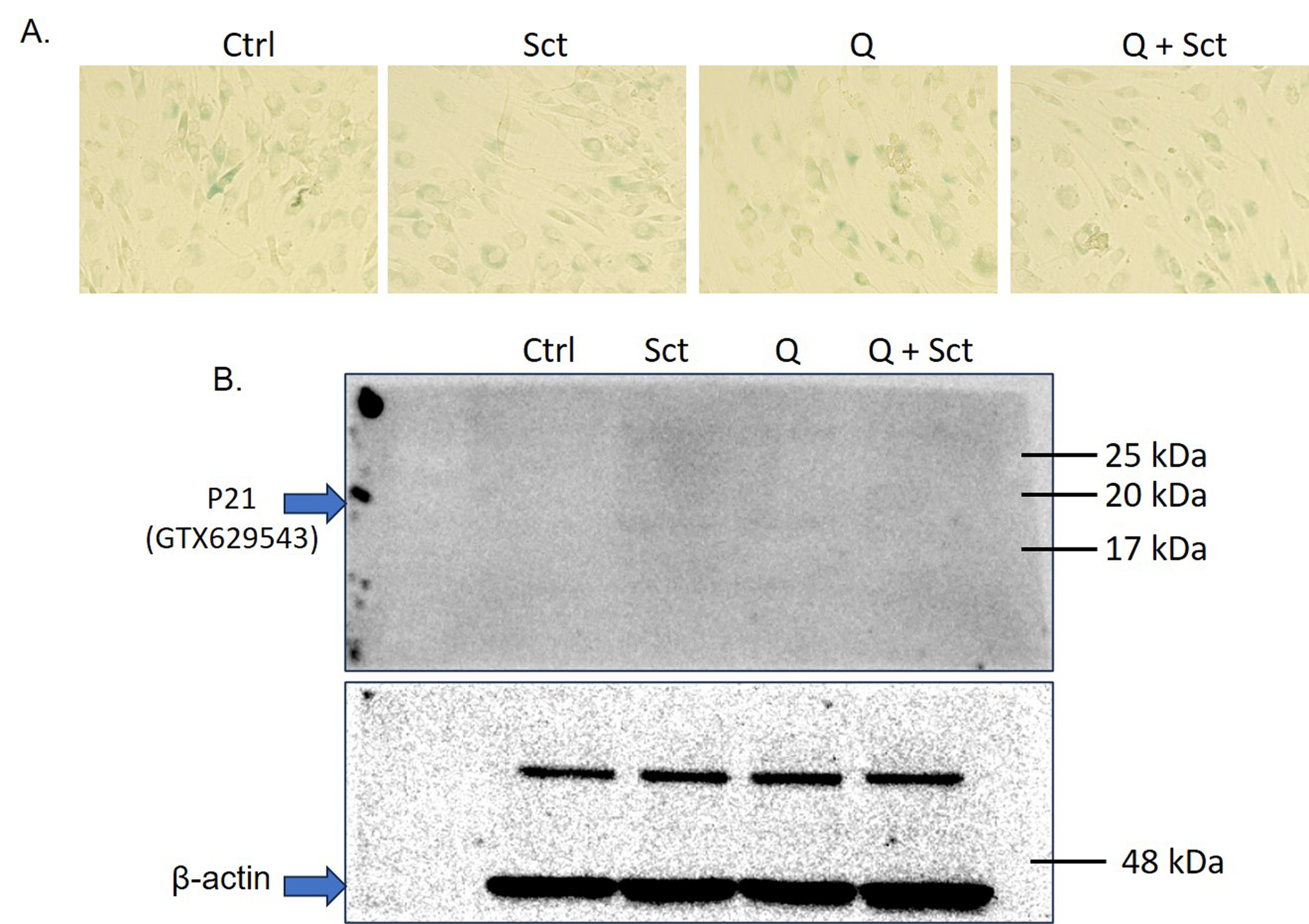

Supplement: Supplementary file 1 [file ijms-26-00281-s001.zip › supplementary figure S1_0.jpg]
